# Interobserver variability between experienced and inexperienced observers in the histopathological analysis of Wilms tumors: a pilot study for future algorithmic approach

**DOI:** 10.1186/s13000-021-01136-w

**Published:** 2021-08-21

**Authors:** Jikke J. Rutgers, Tessa Bánki, Ananda van der Kamp, Tomas J. Waterlander, Marijn A. Scheijde-Vermeulen, Marry M. van den Heuvel-Eibrink, Jeroen A. W. M. van der Laak, Marta Fiocco, Annelies M. C. Mavinkurve-Groothuis, Ronald R. de Krijger

**Affiliations:** 1 Princess Máxima Center for Pediatric Oncology, Heidelberglaan 25, 3584 CS Utrecht, The Netherlands; 2 Utrecht University, Utrecht, The Netherlands; 3 University of Groningen, Groningen, The Netherlands; 4 Department of Pathology, Radboud University Medical Center, Nijmegen, The Netherlands; 5 Mathematical Institute Leiden University, Leiden, The Netherlands; 6 Medical Statistics, Biomedical Data Science Department, Leiden University Medical Center, Leiden, The Netherlands; 7 Department of Pathology, University Medical Center Utrecht, Utrecht, The Netherlands

**Keywords:** Wilms tumor, Interobserver variability, Machine learning, Histopathology, Classification, AI (artificial intelligence)

## Abstract

**Background:**

Histopathological classification of Wilms tumors determines treatment regimen. Machine learning has been shown to contribute to histopathological classification in various malignancies but requires large numbers of manually annotated images and thus specific pathological knowledge. This study aimed to assess whether trained, inexperienced observers could contribute to reliable annotation of Wilms tumor components for classification performed by machine learning.

**Methods:**

Four inexperienced observers (medical students) were trained in histopathology of normal kidneys and Wilms tumors by an experienced observer (pediatric pathologist). Twenty randomly selected scanned Wilms tumor-slides (from *n* = 1472 slides) were annotated, and annotations were independently classified by both the inexperienced observers and two experienced pediatric pathologists. Agreement between the six observers and for each tissue element was measured using kappa statistics (κ).

**Results:**

Pairwise interobserver agreement between all inexperienced and experienced observers was high (range: 0.845–0.950). The interobserver variability for the different histological elements, including all vital tumor components and therapy-related effects, showed high values for all κ-coefficients (> 0.827).

**Conclusions:**

Inexperienced observers can be trained to recognize specific histopathological tumor and tissue elements with high interobserver agreement with experienced observers. Nevertheless, supervision by experienced pathologists remains necessary. Results of this study can be used to facilitate more rapid progress for supervised machine learning-based algorithm development in pediatric pathology and beyond.

## Introduction

Wilms tumors (WTs) account for approximately 90% of all pediatric renal tumors. Since renal tumors account for only 6% of all pediatric malignancies, these tumors are rare [1]. The overall survival of WT patients has increased to 90% in the last four decades [2–4]. Yet*,* there are significant differences in survival within this group of patients, indicating the need for risk stratification. Histological classification is the cornerstone of current risk stratification in WTs, thereby defining individual treatment regimens [5]. However, there is a known interobserver variability, also between experienced pathologists, leading to discrepancies in stage and diagnosis, and thus affecting treatment schedules [6].

Artificial Intelligence (AI) has gained rapidly increasing interest over the past decade. In medicine, recent AI developments show the use of diagnostic algorithms to be contributory to histopathological classification in various malignancies [7–9]. The use of AI techniques (e.g. digital image analysis) might be of additional value in the approach of many tumor types, including WTs, however they rely on supervised machine learning (ML), where computers are trained to recognize specific tissue elements. Large numbers of manually annotated tumor characteristics are required for ML-based classifications, which can be a time-consuming process. Whereas these digital image analysis procedures are emerging in pathology, they are more widely used in radiology [10]. The scarce use in the field of pathology is mainly due to the low level of digitization of the microscopic workflow in many laboratories. With the advent of digital slide scanners, which digitize glass slides into whole slide images (WSIs), digital pathology has now become state-of-the-art in an increasing number of pathology departments [8, 11]. With the increase in digitalization in pathology, we hypothesize that the use of AI in the histopathological classification of WT could find routine implementation and avoid interobserver variability, which could possibly result in more accurate classification of WTs in the future.

To date, no annotated datasets of WT are available and current development of diagnostic algorithms is restricted by the time-consuming process of manual annotations by pathologists. Assistance of specifically trained inexperienced but professional observers in the annotation process for one specific tumor type could potentially aid in the development of diagnostic algorithms. WTs would be suitable as test case, as the tumor has clearly defined triphasic (stromal, blastemal and epithelial) vital tumor components and various chemotherapy-induced changes following preoperative chemotherapy in the setting of the current International Society for Pediatric Oncology (SIOP) treatment protocol [5]. In this study, we aim to assess the interobserver variability of histopathological annotation of WT between two experienced pediatric pathologists (experienced observers) and four trained medical students (inexperienced observers). We hypothesize that medical students can be trained to contribute to the reliable classification of tumor and non-tumor components for further use in the development of ML algorithms for the classification of WT.

## Materials and methods

### Study design and population

This study was performed in a Dutch cohort of 105 WT patients, comprised of all patients < 18 years old with WT in the Netherlands, referred to the Princess Máxima Center for Pediatric Oncology between 2015 and 2019. Diagnosis and classification of WT were done according to the SIOP 2001 and Umbrella SIOP-RTSG 2016 treatment guidelines [5, 12]. All WT patients who gave informed consent for biobanking participation were included. Available Hematoxylin and Eosin (HE) stained slides of this cohort (*n* = 1472) were retrieved from the pathology archives and digitized. The need for ethical approval has been waived by the Medical Ethical Committee (METC 19-314/C). Twenty tumor slides from 20 unique patients were randomly selected for this study. Patient characteristics and tumor classifications of the whole cohort were collected from the nationwide network and registry of histo- and cytopathology in the Netherlands (PALGA).

### Whole Slide Image (WSI) dataset

All 1472 HE slides were digitized at the Radboud University Medical Center (RUMC) using a Pannoramic 1000 digital slide scanner (3DHistech ltd., Budapest, Hungary) at a resolution of 0.24 μm/pixel. Scans were pseudonimized by the RUMC and researchers remained blinded throughout the annotation- and validation process.

### Component selection and annotation

**Fig. 1:**
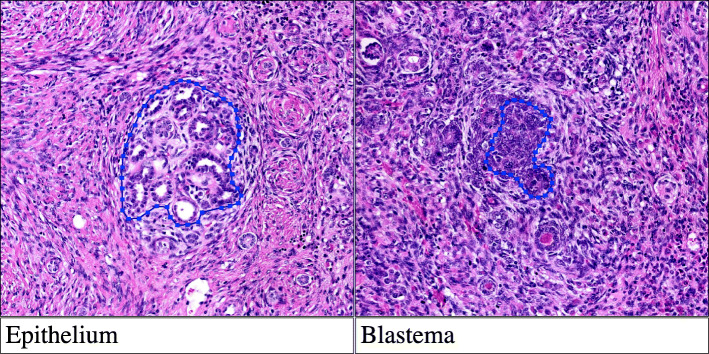
Annotated histopathological features of WT ^a^. ^**a**^ HE stained slide scanned at a 41x equivalent magnification (resolution 0.24 μm/pixel)

Nineteen predefined tissue elements, including vital tumor components, therapy effects and normal tissue elements, were annotated (Table 1; Fig. 1). Element selection was based on expert opinion in consultation with the computational pathology team from the RUMC, as there is no available literature regarding algorithmic approach of WT classification. The annotations and validation were performed using open-source software Automated Slide Analysis Platform (ASAP), version 1.8 [13].

**Table 1:** Annotated tissue elements

**Vital tumor elements**	
1. Blastema	
2. Stroma	
3. Epithelium	
4. Anaplasia	
**Chemotherapy-induced changes**	
5. Necrosis	
6. Bleeding	
7. Regression	
**Normal renal tissue**	
8. Glomeruli	
9. Tubules	
**Extra renal tissue**	
10. Fat	
11. Mesenchyme	
12. Vessels	
13. Nerves	
14. Lymph nodes	
**Adrenal gland**	
15. Adrenal cortex	
16. Adrenal medulla	
**Other**	
17. Urothelium	
18. Nephrogenic rests	
19. Background

### Training

To train the four inexperienced observers, a 2-h histology training by an experienced pediatric pathologist was pursued to recognize each of the selected tissue elements. Following this first training session, the inexperienced observers annotated a test set of ten slides for training. All slides were evaluated extensively and scored in multiple sessions with direct feedback from an experienced pathologist.

### Slide scoring

One of the inexperienced observers annotated approximately 100 annotations in all 20 selected slides. These annotations were encrypted and subsequently labelled to one of the 19 categories by the other observers. All observers remained blinded throughout the process.

### Statistical analysis

Pairwise agreement between the six observers was determined by calculating kappa (κ) statistics. In total 15 values for kappa were estimated, since this is the number of all possible combinations of two individuals in a group of six subjects. In addition, the kappa coefficient of agreement for multiple observers (six) for each category was computed. Here a total of 18 values for kappa were computed. Details about the methodology can be found in Fleiss and Davies [14, 15]. A κ -value greater than 0.8 is considered almost perfect agreement [16]. The statistical analysis was performed in R environment [17].

## Results

### Study population

Twenty WSIs were randomly selected from the total scanned series of 1472 WSIs. These 20 slides were derived from the resection specimens of 20 WT patients with a total of 22 tumors classified according to the SIOP-2001 and the Umbrella SIOP-RTSG 2016 histological classification [5]. The majority was diagnosed with a mixed- or regressive type WT (7 cases each, total of 63.6%) which approximates the histological distribution known from larger cohorts [18]. Table 2 shows an overview of the patient characteristics and tumor types. The mean age at the time of histological diagnosis was 47.5 months (SD 0.47). Contrary to larger cohorts, this cohort shows a larger percentage of females (70.0%) and no epithelial-type tumors, most likely due to the small sample size and its resulting bias.

**Table 2:** Patient characteristics and tumor classification

**Patient characteristics (*n*** **= 20)**	
Age in months at time of diagnosis, *mean (SD)*	47.5 (0.47)
Male gender, *n (%)*	6/20 (30.0)
Right-sided WT localization, *n (%)*	11/20 (55.0)
Primary resection, *n (%)*	2/20 (10.0)
Lymph node metastases, *n (%)*	2/19 (10.5)
**Tumor classification (*n*** **= 22)**	**n (%)**
Tumor type^a, b^	
*Low Risk*	
Completely necrotic	2 (9.1)
*Intermediate risk*	
Non-anaplastic variants^c^	2 (9.1)
Epithelial type	–
Stromal type	1 (4.5)
Mixed type	7 (31.8)
Focal anaplasia	1 (4.5)
Regressive type	7 (31.8)
*High risk*	
Blastemal type	1 (4.5)
Diffuse anaplasia	1 (4.5)

^a^ WT stratification according to the Nephroblastoma Umbrella SIOP-RTSG 2016 pathology guidelines [5]. ^b^ *N* = 22 due to presence of multiple (two) tumors in two cases, which are classified individually. ^c^ In primary nephrectomy cases only

### Interobserver variability

**Fig. 2:**
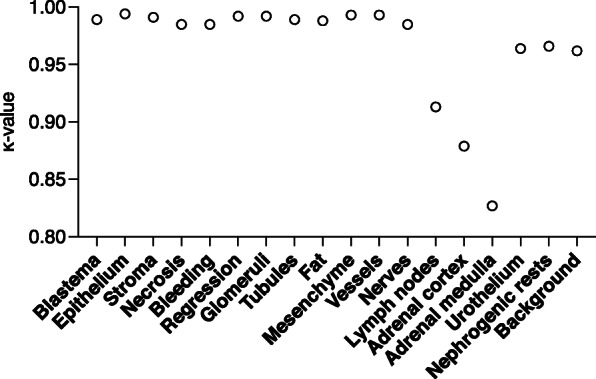
Interobserver agreement per annotated tissue element for all six observers

The pairwise interobserver agreement between all inexperienced and experienced observers ranged from 0.845 to 0.950, Table 3. The interobserver variability estimated of each histological element separately showed all κ-coefficients being > 0.827, Fig. 2. The tumor elements relevant for classification (i.e. blastema, epithelium, stroma and chemotherapy-induced changes) had an interobserver agreement that ranged from 0.985 to 0.994. Adrenal gland elements had a slightly lower agreement with a κ statistic of respectively 0.827 and 0.879 for adrenal medulla and adrenal cortex.

**Table 3:** Pairwise agreement κ between observers (approximate significance) ^a^

	P1	P2	S1	S2	S3	S4
**P1**		.948 (0.005)	.938 (0.006)	.928 (0.006)	**.**944 (0.005)	.877 (0.008)
**P2**	.948 (0.005)		.928 (0.006)	.929 (0.006)	.938 (0.006)	.866 (0.008)
**S1**	.938 (0.006)	.928 (0.006)		.915 (0.007)	.921 (0.006)	.872 (0.008)
**S2**	.928 (0.006)	.929 (0.006)	.915 (0.007)		.950 (0.005)	.845 (0.009)
**S3**	.944 (0.005)	.938 (0.006)	.921 (0.006)	.950 (0.005)		**.**866 (0.008)
**S4**	.877 (0.008)	.866 (0.008)	.872 (0.008)	.845 (0.009)	**.**866 (0.008)	

^**a**^ P1 and P2: experienced observers (pathologists); S1, S2, S3 and S4: inexperienced observers (trained medical students)

We were not able to assess a κ statistic of the WT component ‘anaplasia’, since no (diffuse) anaplasia was identified in the selected WSIs by both the inexperienced and experienced observers.

## Discussion

The results suggest that trained, inexperienced observers, such as medical students, in the histopathological annotation of WT components and non-tumoral elements may help in the classification of histological elements. This information can be used for the development of diagnostic algorithms. However, this does not mean that experienced pathologists can be replaced by inexperienced observers, even after training.

The initial selection (annotation) is a time-consuming process, and trained students could potentially aid in the development of large annotated datasets to train diagnostic algorithms for pediatric tumors. The vast majority of annotations in this study showed a very good agreement, however fine-tuning and corrections by an expert pediatric pathologist remain essential to train the model.

The three vital tumor elements of WT; stroma, blastema, and epithelium, are of great importance in the risk stratification of WTs. Pathologists determine the percentage of each category by estimating the integrated relative contribution to the total vital tumor area of all slides. After pre-operative chemotherapy, the assessed percentage of blastema in the remaining vital part of the tumor, has a direct consequence for the treatment of the patient. Central review has shown that the interobserver variability, even among experienced pathologists, might lead to moderately reproducible values, as analysis is based on eyeballing and percentages are not formally calculated. Hermsen et al. developed a deep-learning algorithm for renal tissue which showed to be capable of very precise segmentation of specific structures (e.g. glomeruli, tubules) of whole nephrectomy specimens [7]. The good interobserver agreement in our study shows that inexperienced observers are able to learn to identify the different histological elements of WT.

The three elements representing therapy effect do also influence risk stratification and treatment regimen, similar to vital tumor elements. This is especially the case in (near) complete necrosis, where no postoperative chemotherapy is required, or in tumors with 65–70% regression and a dominant blastemal component in the vital tumor area, which may or may not end up in the high-risk group [19]. All three therapy effect elements showed satisfying interobserver agreement in our study. The discrepancies between observers were mostly related to the distinction of vital stromal tissue from paucicellular areas of regression. While cellularity and relationship to other vital tumor components or to other areas with regressive features may give clues to the correct classification; this distinction is difficult, even for experienced observers [6]. However, this did not lead to an unsatisfactory interobserver variability, against the background of previous training sessions.Interestingly, the interobserver variability of nephrogenic rests is, in line with all other tissue components, remarkably good (κ statistic 0.966). Nephrogenic rests are pre-malignant, abnormal residual clusters of embryonal cells of the developing kidney and can be located in the peripheral renal cortex (perilobar nephrogenic rests) as well as within the renal parenchyma (intralobar nephrogenic rests) [20]. These clusters can present similar architecture to vital epithelial structures of WT and even, when hyperplastic, show similarities to blastemal WT cells. These histopathological similarities, together with the variable localization of the nephrogenic rests, make recognition even challenging for expert pathologists [21]. The current high agreement might be explained by the fact that in this study most areas that were classified as nephrogenic rests by one of the experienced pathologists, were classified as vital WT epithelium by the other observers. This leads to low interobserver variability, even while misclassifying this category with respect to the opinion of the experienced observer, which is an inherent weakness of any interobserver variability study. Additionally, nephrogenic rests were not present in all slides, resulting in a low number of annotations. To identify nephrogenic rests correctly, supervision by experienced pathologists is required. The data of the other annotation categories were checked whether the high interobserver agreement resulted from misclassification as opposed to expert classification, but except for nephrogenic rests, this phenomenon was not found for any of the other tissue categories.

There are some limitations to this study. Firstly, only 20 slides have been used. However, as each slide approximates 100 annotations, this does lead to a substantial number (*n* = 1976) of annotations resulting in an interobserver agreement based on 1976 comparisons. Focal nor diffuse anaplasia was present in the selected slides and was therefore excluded from the analysis.

Another issue is the fact that annotations have initially been set by one of the unexperienced observers, potentially leading to selection bias. While this might have been the case, the number of annotations and the equal distribution over the various categories has ensured sufficient representation of relevant images.

## Conclusion

Inexperienced observers can be trained to recognize specific histopathological tumor and tissue elements with a high interobserver agreement with expert pediatric pathologists and among inexperienced observers. Therefore, this study can serve as the basis for further development of automated component analysis of pretreated WT by an ML-based algorithm, which may lead to more accurate and reproducible risk group classification of WT [7].

## Data Availability

The dataset of this study concerns a small number of anonymized cases with limited data that are available to any interested reader upon request.

## Abbreviations

WT: Wilms tumors
AI: Artificial Intelligence
ML: Machine learning
WSIs: Whole slide images
SIOP: International Society for Pediatric Oncology
HE-slides: Hematoxylin and Eosin stained slides
Κ: Kappa
